# Genetic Characteristics of Human Parainfluenza Virus Types 1–4 From Patients With Clinical Respiratory Tract Infection in China

**DOI:** 10.3389/fmicb.2021.679246

**Published:** 2021-07-15

**Authors:** Nan Shao, Bo Liu, Yan Xiao, Xinming Wang, Lili Ren, Jie Dong, Lilian Sun, Yafang Zhu, Ting Zhang, Fan Yang

**Affiliations:** ^1^NHC Key Laboratory of Systems Biology of Pathogens, Institute of Pathogen Biology, Chinese Academy of Medical Sciences and Peking Union Medical College, Beijing, China; ^2^NHC Key Laboratory of Systems Biology of Pathogens and Christophe Mérieux Laboratory, Institute of Pathogen Biology, Chinese Academy of Medical Sciences and Peking Union Medical College, Beijing, China; ^3^Key Laboratory of Respiratory Disease Pathogenomics, Chinese Academy of Medical Sciences and Peking Union Medical College, Beijing, China

**Keywords:** human parainfluenza virus, hemagglutinin-neuraminidase gene, fusion gene, phylogenetic analysis, recombination analysis, glycosylation site

## Abstract

Human parainfluenza viruses (HPIV1–4) cause acute respiratory tract infections, thereby impacting human health worldwide. However, there are no current effective antivirals or licensed vaccines for infection prevention. Moreover, sequence information for human parainfluenza viruses (HPIVs) circulating in China is inadequate. Therefore, to shed light on viral genetic diversity and evolution, we collected samples from patients infected with HPIV1–4 in China from 2012 to 2018 to sequence the viruses. We obtained 24 consensus sequences, comprising 1 for HPIV1, 2 for HPIV2, 19 for HPIV3, and 2 for HPIV4A. Phylogenetic analyses classified the 1 HPIV1 into clade 2, and the 2 HPIV4 sequences into cluster 4A. Based on the hemagglutinin-neuraminidase (HN) gene, a new sub-cluster was identified in one of the HPIV2, namely G1c, and the 19 HPIV3 sequences were classified into the genetic lineages of C3f and C3a. The results indicated that HPIV1–4 were co-circulated in China. Further, the lineages of sub-cluster C3 of HPIV3 were co-circulated in China. A recombination analysis indicated that a putative recombination event may have occurred in the HN gene of HPIV3. In the obtained sequences of HPIV3, we found that two amino acid substitution sites (R73K in the F protein of PUMCH14028/2014 and A281V in the HN protein of PUMCH13961/2014) and a negative selection site (amino acid position 398 in the F protein) corresponded to the previously reported neutralization-related sites. Moreover, amino acid substitution site (K108E) corresponded to the negative selection site (amino acid position 108) in the 10 F proteins of HPIV3. However, no amino acid substitution site corresponded to the glycosylation site in the obtained HPIV3 sequences. These results might help in studying virus evolution, developing vaccines, and monitoring HPIV-related respiratory diseases.

## Introduction

Human parainfluenza viruses (HPIVs) belong to the family Paramyxoviridae. HPIVs are enveloped, non-segmented, single-stranded, negative-sense RNA viruses with a genome length of 14.9–17.3 kb that encodes six common structural proteins, namely N, P, M, fusion (F), hemagglutinin-neuraminidase (HN), and L. Of these, F and HN glycoproteins are membrane-associated proteins, and N, P, and L, are nucleocapsid-associated proteins (Karron and Collins, 2013). HPIVs have been grouped into four serotypes according to genetic and antigenic variation (HPIV1–4), among which HPIV1 and HPIV3 are classified into the genus *Respirovirus*, whereas HPIV2 and HPIV4 (subtypes HPIV4A, and HPIV4B) are classified into the genus *Rubulavirus* (Canchola et al., 1964; Henrickson, 2003).

Human parainfluenza viruses are important causative agents of acute respiratory infections (ARIs) and commonly cause upper and lower respiratory tract infections (Schomacker et al., 2012). Lower respiratory tract infections are considered the principal cause of hospitalization and death in certain groups of people (Marx et al., 1999; Mao et al., 2012; Seo et al., 2014). The clinical symptoms of respiratory tract infections include croup, bronchitis, bronchiolitis, and pneumonia; however, some clinical manifestations are more related to individual HPIVs. HPIV1 and HPIV2 are most likely cause croup, whereas HPIV3 and HPIV4 are more associated with bronchiolitis and pneumonia (Fathima et al., 2016; Linster et al., 2018). Furthermore, HPIVs can co-infect with other viruses or bacteria, such as influenza viruses A and B, human respiratory syncytial virus (RSV), human rhinovirus, adenovirus, human coronavirus, human metapneumovirus, *Mycoplasma pneumoniae*, and *Chlamydia* (Liu et al., 2013; Zhong et al., 2019).

Moreover, HPIVs are distributed worldwide and threaten vulnerable people of all ages, as well as those who are immunocompromised or have undergone hematopoietic stem cell transplantation (Iwane et al., 2004; Ustun et al., 2012; Shi et al., 2015). A previous population-based surveillance report showed HPIVs are second to RSV in causing ARI-related hospitalizations in children under 5 years old, with higher admissions rates for children under 1 year old (Weinberg et al., 2009; Morgan et al., 2013; Goya et al., 2016; Xiao et al., 2016). HPIV infection rates are the highest in children under 5 years old, followed by patients over 60 years old (Pan et al., 2017). In addition, HPIV1–4 have been detected in China, and HPIV3 was the predominant type of HPIVs epidemic in China (Lau et al., 2009; Ren et al., 2009, 2011; Wang et al., 2015; Xiao et al., 2016; Li C. X. et al., 2020; Li H. J. et al., 2020). Notably, HPIV infections do not induce complete protective immunity, and specific antiviral drugs and licensed vaccines are not yet available for the treatment and prevention of HPIV infections (Henrickson, 2003). The economic burden attributed to the morbidity and mortality of HPIV-associated hospitalization cannot be ignored.

The complete genome information available for HPIV1–4 in the GenBank database is currently insufficient. Most sequences are for the HN gene, and sequences are biased toward certain countries. Overall, the sequence information for HPIV1–4 from China available in public databases is limited. In China, there are no complete genomes for HPIV1 and HPIV2, the complete genomes for HPIV3 are few, and the two complete genomes of HPIV4A are only from Taiwan at present. Besides, a recombination event with a breakpoint at the N gene of HPIV3 has also been identified (Yang H. T. et al., 2011). Therefore, a clear analysis of the homologous recombination events in HPIVs from human hosts is essential.

More sequence information about HPIV1–4 is thus required to fully understand their genetic diversity, evolution and potential recombination events, which are conducive to in-depth monitoring, prevention, and control of pathogens. In addition, there should be a focus on the glycosylation site, selection site, amino acid substitution site and neutralization-related amino acid site, which are critical to viral antigenicity and biological activity. Therefore, we used both traditional Sanger sequencing and next generation sequencing (NGS; Illumina, MiSeq) to obtain the sequence information of HPIVs from patients associated with respiratory tract infections in this study.

## Materials and Methods

### Ethics Statement

This study was performed with strict accordance with the human subject protection guidance. Moreover, this study was approved by the Ethics Committee of the Institute of Pathogen Biology, Chinese Academy of Medical Sciences and Peking Union Medical College, and the Ethics Committee of Hospitals. The written informed consent was obtained from the patients or the participants’ parents or guardians.

### Clinical Sample and Multiplex PCR Detection

Clinical samples including oropharyngeal and nasopharyngeal swabs, as well as deep sputum were collected from 24 inpatients and outpatients with respiratory tract infections in six cities in China. Sample viral nucleic acids were extracted using the NucliSens easyMAG apparatus (bioMérieux, Marcy l’Etoile, France) in BSL-2 as previously described (Xu et al., 2017). All the 24 clinical samples were identified to be positive for HPIVs. Of these, 16 HPIV-positive specimens (HPIV3 and HPIV4) collected in 2012 and 2014 were examined by a multiplex RT-Nested polymerase chain reaction (PCR) described previously (Coiras et al., 2004), and eight HPIV-positive specimens (HPIV1, HPIV2, and HPIV3) collected in 2017 and 2018 were tested by an ABI 7500 Fast.

### Double-Stranded cDNA Synthesis From Viral Nucleic Acids

Viral nucleic acids extracted from the 24 samples were used to synthesize first-strand complementary DNA (cDNA) using a SuperScript^TM^ IV First-Strand Synthesis system (Invitrogen, United States) with 100 pM of primer K-8N (5′-GACCATCTAGCGACCTCCAC-NNNNNNNN-3′). The cDNA was further converted into double-stranded cDNA (ds cDNA) with a Klenow fragment (NEB, United States) at 37°C for 1 h and at 75°C for 10 min.

### PCR Amplification and Sanger Sequencing

The genome sequences obtained from the eight positive specimens collected in 2017 and 2018 were amplified by primers designed using the Primer 5.0 software according to the full-length of the HPIV1, HPIV2, and HPIV3 reference genomes. The total reaction volume was 50 μL, including 1 μL ds cDNA, 1 μL of each primer, 25 μL Premix Taq^TM^ (Takara, Japan), and 23 μL nuclease-free water. The thermal cycling conditions were as follows: 94°C for 5 min, 40 cycles of 94°C for 30 s, 50°C for 30 s, 72°C for 2 min, and then 72°C for 10 min. The PCR products were sequenced by the Sanger method.

### PCR Amplification and NGS Sequencing

The entire genome sequences obtained from the 16 positive samples collected in 2012 and 2014 were amplified using six sets of universal primers corresponding to HPIV3 and HPIV4 (Supplementary Table 1). The first round PCR was performed in a total reaction volume of 50 μL, including 0.5 μL of ds cDNA, 25 μL of Premix Taq^TM^ (Takara, Japan), 1 μL of forward and reverse primers (10 pmol/μL for each primer), and 22.5 μL of nuclease-free water. The amplification reaction comprised 30 cycles. A second round of PCR comprising 40 cycles, was performed in a total reaction volume of 50 μL, including 0.5 μL templates from the first round PCR amplification products. Then, the second round PCR products were purified via a QIAquick PCR Purification Kit (Qagien, Hilden, Germany) and prepared for NGS sequencing.

The purified PCR products of each sample were mixed at equal molar concentrations. Libraries were constructed using the Nextera^®^ XT Library Prep Kit (Illumina, San Diego, CA, United States) according to the manufacturer’s instructions. AMPure XP beads were used to clean up the libraries, and the sizes of fragments were assessed by an Agilent 2100 bioanalyzer system (Thermo Fisher). Pooled libraries involving 24 samples were sequenced by a 600 cycle Miseq Reagent kit (v3) (Illumina, San Diego, CA, United States) according to the manufacturer’s recommendations at a length of 300 bp paired-end reads.

### Genome Assembly

Raw sequence reads were filtered using the previously described criteria (Yang J. et al., 2011; Liu et al., 2020) to obtain valid sequence reads, which were exported using Megan (ver. 6) MetaGenome Analyzer (Huson et al., 2016). Valid reads from NGS were assembled and manually edited using the Lasergene SeqMan program (DNASTAR, Madison, WI, United States). The longest contig was then compared with the sequences available in the GenBank database to select the closest reference sequence. The sequencing reads from Sanger were trimmed using BioEdit software (Tom Hall, North Carolina State University, Carolina) and the closest reference sequence was found using the GenBank database. All reads were mapped to individual reference sequences, and any low-quality reads were manually curated. Additionally, targeted PCR reactions were also performed to process the remaining gene gaps to improve genome coverage when necessary.

### Identity Analysis

Publicly available HPIV sequences were downloaded from the GenBank database on August 13, 2020, and variants, clones, modified microbial nucleic acids, synthetic constructs, virus-like particles, non-human host viruses, and sequences with accidental codon insertions or most base deletions were excluded. Sequences that could be distinguished from most other sequences due to base insertions were eliminated. Sequences of HPIVs (types 1–4) were aligned with their respective obtained sequences. Multiple nucleotide sequence alignments were analyzed using the ClustalX (ver. 2.0) (Larkin et al., 2007) and Clustal Omega programs (Sievers and Higgins, 2018). The percent identity of the nucleotide and deduced amino acid sequences was assessed using MegAlign (Lasergene v.7.0.1).

### Phylogenetic Analysis and Evolutionary Divergence Estimation

Phylogenetic trees based on the complete coding sequence (CDS) of the F and HN genes were constructed with the maximum likelihood method using Mega software (ver. 5.05) (Tamura et al., 2011). An optimal nucleotide substitution model of Tamura-Nei distances and a gamma distribution with invariant sites (G + I) were recommended by the model function in the program MEGA, and the robustness of the phylogenetic analysis was assessed using 1,000 bootstrap replicates. The between-group evolutionary divergences (involving clusters, subclusters, and genetic lineages) of the F and HN gene sequence pairs of HPIV3 were estimated using MEGA 5.05. The number of base substitutions per site from averaging over all sequence pairs between groups were estimated using the Tamura-Nei model with 1,000 bootstrap replicates (Tamura and Nei, 1993).

### Recombination Analysis

RDP5 software^1^ and Simplot version 3.5.1^2^ were used for putative recombination analysis. RDP5 was firstly used to examine recombination signal with the default parameters using different detection methods, including RDP, GENECONV, MaxChi, BootScan, and SisScan. Then, the putative recombination sequence detected by RDP5 was examined in the Simplot program through similarity and bootscaning analysis with a sliding window size of 200 bp and a moving step size of 20 bp.

### Glycosylation Site Analysis

The amino acid sequences of the complete F and HN genes of HPIV1–4 were used for *N*-glycosylation and GalNAc *O*-glycosylation site prediction using the NetNGlyc 1.0 and 4.0 server, respectively^3^. Sites with threshold scores of higher than 0.5 were classified as glycosylated.

### Selection Sites Analysis

Positive and negative selection sites on the F and HN gene of HPIV3 were estimated using the Datamonkey web server^4^. The Mixed Effects Model of Evolution (MEME) was used to evaluate positive selection sites and the Fixed Effects Likelihood (FEL) model was used to estimate negative selection sites. Significance level for both the MEME and FEL methods were a p-value threshold less than 0.05.

### Amino Acid Substitutions Analysis

The locations of all amino acid substitutions in the F and HN proteins of the sequenced HPIV1–4 were identified using the corresponding individual prototype strains. To further clarify the relationships among amino acid substitution sites, negative selection sites and glycosylation sites in the F and HN proteins of the sequenced HPIV3, and the previously reported neutralization-related amino acid sites, structural models were constructed using SWISS-MODEL^5^ (Waterhouse et al., 2018) with individual prototype strains. For sequences of the F protein of HPIV3, the positions of amino acid substitutions were mapped to the prototype strain Wash/47885/57 (GenBank accession number: S82195), and homology modeling was constructed according to the crystal structure of 1ZTM (Protein Data Bank ID). For sequences of the HN protein of HPIV3, the positions of amino acid substitution sites were mapped to the prototype strain Washington 1957 (GenBank accession number: JN089924), homology modeling was employed by the crystal structure of 4MZE (Protein Data Bank ID). Related sites were labeled on structural figures using PyMOL (Schrödinger).

## Results

### Detection and Sequencing Analysis

The clinical information of the 24 patients from six cities is shown in Table 1. In total, one HPIV1, two HPIV2, 19 HPIV3, and two HPIV4 sequences were obtained from the clinical samples that collected in 2012, 2014, 2017, and 2018. The age of the patients ranged from 2 months to 90 years, and presented a wide spectrum of clinical characteristics. The reads from the Sanger method and valid reads from NGS were assembled using the SeqMan program according to individual references (GenBank accession numbers: AF457102, MH892405, EU326526, and KY460518 for HPIV1, HPIV2, HPIV3, and HPIV4A, respectively). Herein, the one HPIV1 and two HPIV4A sequneces had a complete CDS, and the complete CDS coverage of the two HPIV2 both exceeded 97%. The 19 HPIV3 sequences included seven complete CDS, three partial genes (51–99% complete CDS coverage), and nine full-length HN genes, that is, 10 F genes and 19 HN genes of HPIV3 have been obtained (see Table 2).

**TABLE 1:** Information about HPIV1–4 identified in this study.

**Case ID**	**Lab number**	**Type**	**Gender**	**Age**	**Location**	**Collection date**	**Source**	**In/out patient**	**Clinical characteristics**	**Sequencing method**
**1**	JL18058d5	HPIV1	Male	71 years	Jilin	15-March-2018	Deep sputum	In	Fever, cough, expectoration	Sanger
**2**	WH17030d1	HPIV2	Male	70 years	Wuhan	21-October-2017	Deep sputum	In	Cough, expectoration	Sanger
**3**	WH17080d3	HPIV2	Male	60 years	Wuhan	20-November-2017	Deep sputum	Out	Cough, expectoration	Sanger
**4**	BCH-4100A	HPIV3	Male	9 months 15 days	Beijing	04-May-2014	Nasopharyngeal swab	In	Pneumonia, respiratory failure	NGS + Sanger
**5**	BCH-4102A	HPIV3	Male	6 months 13 days	Beijing	03-May-2014	Nasopharyngeal swab	In	Acute pneumonia	NGS + Sanger
**6**	BCH-4210A	HPIV3	Female	1 year 2 months 28 days	Beijing	14-July-2014	Nasopharyngeal swab	In	Bronchopneumonia	NGS + Sanger
**7**	PUMCH12693	HPIV3	Female	76 years	Beijing	30-May-2012	Pharyngeal swab	Out	Upper respiratory tract infection	NGS + Sanger
**8**	PUMCH13998	HPIV3	Female	46 years	Beijing	30-Apr-2014	Pharyngeal swab	Out	Upper respiratory tract infection	NGS + Sanger
**9**	PUMCH14028	HPIV3	Female	22 years	Beijing	15-June-2014	Pharyngeal swab	Out	Upper respiratory tract infection	NGS + Sanger
**10**	BCH-3297A	HPIV3	Male	2 months 16 days	Beijing	25-July-2012	Nasopharyngeal swab	In	Pneumonia	NGS + Sanger
**11**	BCH-4138A	HPIV3	Male	2 years 11 days	Beijing	27-May-2014	Nasopharyngeal swab	In	Acute pneumonia	NGS + Sanger
**12**	BCH-3242A	HPIV3	Female	4 months 21 days	Beijing	23-May-2012	Nasopharyngeal swab	In	Pneumonia, congenital heart disease, respiratory failure	NGS + Sanger
**13**	BCH-4066A	HPIV3	Male	4 months 20 days	Beijing	16-April-2014	Pharyngeal swab	In	Pneumonia, diarrhea	NGS + Sanger
**14**	PUMCH12726	HPIV3	Male	90 years	Beijing	06-June-2012	Pharyngeal swab	Out	Upper respiratory tract infection	Sanger
**15**	PUMCH12885	HPIV3	Female	55 years	Beijing	17-July-2012	Pharyngeal swab	Out	Upper respiratory tract infection	Sanger
**16**	PUMCH13961	HPIV3	Female	38 years	Beijing	10-April-2014	Pharyngeal swab	Out	Upper respiratory tract infection	NGS + Sanger
**17**	BCH-3283A	HPIV3	Female	1 month 22 days	Beijing	04-July-2012	Nasopharyngeal swab	In	Pneumonia, respiratory failure	NGS + Sanger
**18**	HB16067d9	HPIV3	Male	54 years	Harbin	24-January-2017	Deep sputum	In	Fever, cough, expectoration, shortness of breath	Sanger
**19**	WH17001d3	HPIV3	Male	37 years	Wuhan	11-April-2017	Deep sputum	Out	Fever, cough, expectoration	Sanger
**20**	XA17013d1	HPIV3	Male	53 years	Xian	18-May-2017	Deep sputum	In	Fever, cough, expectoration	Sanger
**21**	CD17001d7	HPIV3	Male	34 years	Chengdu	06-September-2017	Deep sputum	In	Expectoration, shortness of breath, fatigue	Sanger
**22**	CD17004d3	HPIV3	Male	16 years	Chengdu	29-September-2017	Deep sputum	Out	Miss information	Sanger
**23**	BCH-4237A	HPIV4	Male	1 year 2 days	Beijing	11-August-2014	Nasopharyngeal swab	In	Pneumonia	NGS + Sanger
**24**	BCH-4263A	HPIV4	Male	2 months	Beijing	01-September-2014	Nasopharyngeal swab	In	Bronchial asthma	NGS + Sanger

**TABLE 2:** Sequences information of the obtained HPIV1–4 sequences.

**Lab number**	**Types**	**Genes**	**Accession numbers**
JL18058d5/2018	1	Complete CDS	MW575643
WH17030d1/2017	2	Partial N, P, M, F, HN, partial L	MW575644
WH17080d3/2017	2	Partial N, P, M, F, HN, partial L	MW575645
BCH4100A/2014	3	Complete CDS	MW575653
BCH4102A/2014	3	Complete CDS	MW575654
BCH4210A/2014	3	Complete CDS	MW575655
PUMCH12693/2012	3	Complete CDS	MW575656
PUMCH13988/2014	3	Complete CDS	MW575657
PUMCH14028/2014	3	Complete CDS	MW575658
BCH3297A/2012	3	Complete CDS	MW575659
BCH4138A/2014	3	N, P, M, F, HN, partial L	MW575660
BCH3242A/2012	3	N, P, M, F, HN, partial L	MW575661
BCH4066A/2014	3	N, partial P, M, F, HN	MW575662
PUMCH12726/2012	3	HN	MW575646
PUMCH12885/2012	3	HN	MW575647
PUMCH13961/2014	3	HN	MW575663
BCH3283A/2012	3	HN	MW575664
HB16067d9/2017	3	HN	MW575648
WH17001d3/2017	3	HN	MW575649
XA17013d1/2017	3	HN	MW575650
CD17001d7/2017	3	HN	MW575651
CD17004d3/2017	3	HN	MW575652
BCH4237A/2014	4A	Complete CDS	MW575665
BCH4263A/2014	4A	Complete CDS	MW575666

### Identity Analysis

The nucleotide and amino acid identities of the complete CDS were calculated. For HPIV1–4 sequences obtained in this study, the computed values of the nucleotide and amino acid identities were the same. The nucleotide identities between the obtained HPIV1 sequence and the downloaded HPIV1 sequences were 94.4–98.7%, and those between the two obtained HPIV2 sequences and the downloaded HPIV2 sequences were 91.5–96.2% and 91.3–95.9%, respectively. The nucleotide identities between the downloaded and obtained sequences of HPIV3 ranged from 93.8% to 99.5%. The nucleotide identities of the two obtained HPIV4A sequences and the downloaded HPIV4A sequences were 96.9–98.9% and 96.8–98.5%, respectively (see Table 3).

**TABLE 3:** The nucleotide and amino acid identities of HPIV1–4 based on the complete CDS.

**Lab number**	**Comparison within types**	**Nucleotide identity (%)**	**Amino acid identity (%)**
JL18058d5/2018	HPIV1	94.4–98.7	94.4–98.7
WH17030d1/2017	HPIV2	91.5–96.2	91.5–96.2
WH17080d3/2017	HPIV2	91.3–95.9	91.3–95.9
BCH4100A/2014	HPIV3	94.2–99.3	94.2–99.3
BCH4102A/2014	HPIV3	94.0–99.5	94.0–99.5
BCH4138A/2014	HPIV3	93.8–99.4	93.8–99.4
BCH4210A/2014	HPIV3	94.1–99.4	94.1–99.4
PUMCH12693/2012	HPIV3	94.2–99.3	94.2–99.3
PUMCH13988/2014	HPIV3	94.1–99.5	94.1–99.5
PUMCH14028/2014	HPIV3	94.0–99.5	94.0–99.5
BCH3242A/2012	HPIV3	93.8–99.0	93.8–99.0
BCH3297A/2012	HPIV3	94.2–99.3	94.2–99.3
BCH4237A/2014	HPIV4A	96.9–98.9	96.9–98.9
BCH4263A/2014	HPIV4A	96.8–98.5	96.8–98.5

### Phylogenetic Analysis of the F Gene and Evolutionary Divergence Estimation

**FIGURE 1:**
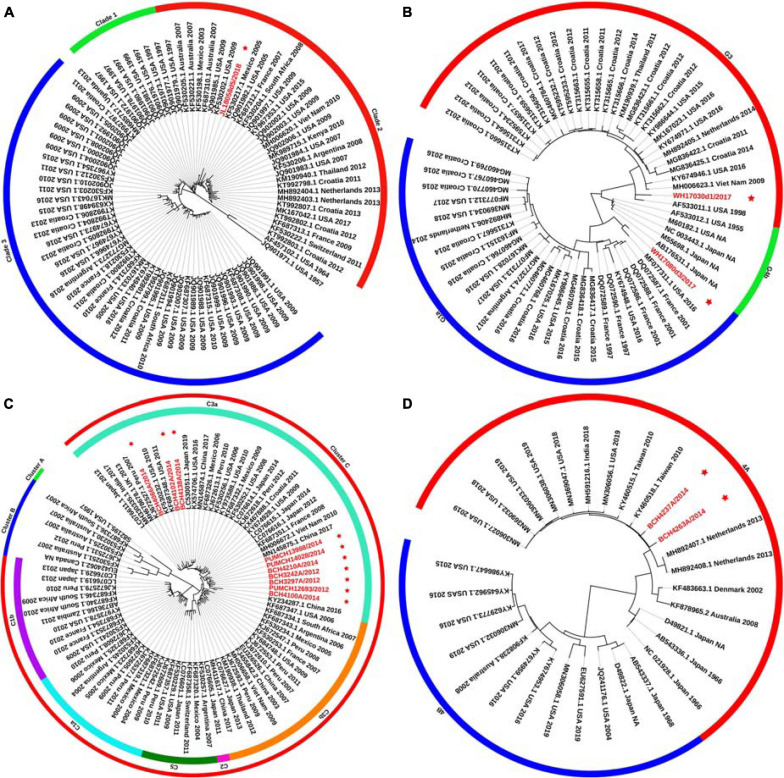
Phylogenetic tree based on the entire F gene of HPIV1–4 and labels marked with a red star indicated the sequences identified in this study. Panels **(A–D)** correspond to the trees of HPIV1, HPIV2, HPIV3, and HPIV4, respectively. Genetic classification was distinguished by colored strips. NA, not available.

Phylogenetic trees for the F gene (numbers = 85, 60, 85, and 30 for HPIV1, HPIV2, HPIV3, and HPIV4, respectively) were constructed (Figure 1). The distribution of the HPIV1 F gene indicated that the identified HPIV1 belonged to clade 2 and was closed to viruses from the United States identified in 2009. The two obtained HPIV2 sequences, WH17030d1/2017 and WH17080d3/2017, were segregated into cluster G3 and G1a, respectively, and closed to the viruses from the United States identified in 2016. Ten of the obtained HPIV3 sequences were classified into cluster C3, and were close to the viruses from the United States, Japan, and Vietnam. The two obtained HPIV4A sequences were close to the viruses from Taiwan identified in 2010. For the 85 F gene sequences of HPIV3, the estimated values of evolutionary distance and standard error are shown in Supplementary Table 2. Of these, the divergence value between the genetic lineages C3a and C3b was 0.018 ± 0.002.

### Phylogenetic Analysis of the HN Gene and Evolutionary Divergence Estimation

**FIGURE 2:**
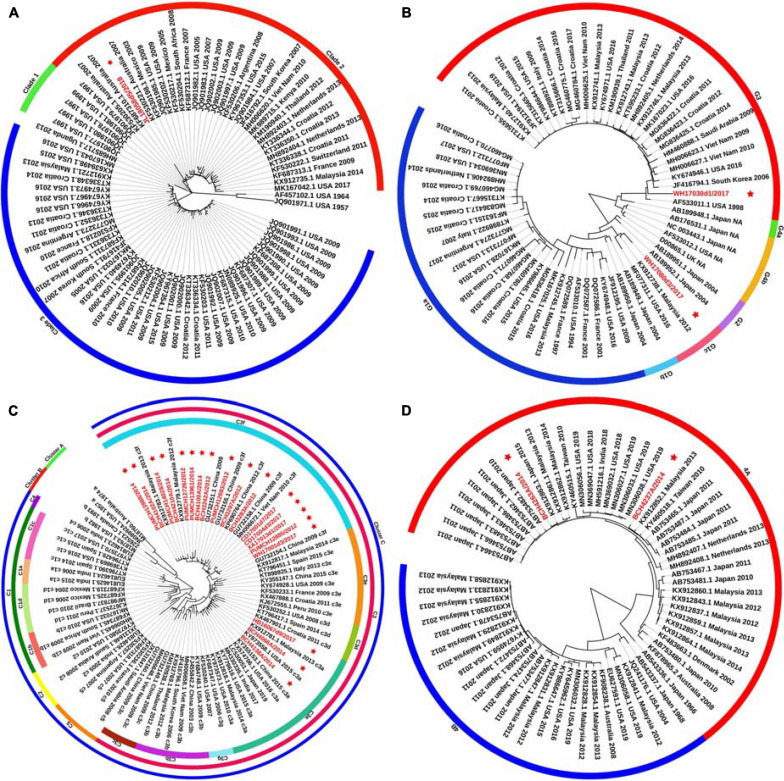
Phylogenetic tree based on the full-length HN gene of HPIV1–4 and labels marked with a red star indicated the sequences identified in this study. Panels **(A–D)** correspond to the trees of HPIV1, HPIV2, HPIV3, and HPIV4, respectively. Genetic classification was distinguished by colored strips. NA, not available.

Phylogenetic trees were constructed based on the HN gene (numbers = 82, 65, 83, and 65 for HPIV1–4, respectively) (Figure 2). The newly identified HPIV1 (JL18058d5/2018) virus was grouped into clade 2, and was close to HPIV1/MEX/495/2003 and HPIV1/AUS/54/2007. The two HPIV2 sequences obtained herein were grouped into two distinct clusters. WH17030d1/2017 was grouped into cluster G3, formed an independent lineage, and was close to strains circulating in the United States and South Korea. WH17080d3/2017 was grouped into a new sub-cluster G1c and was close tostrains circulating in Malaysia and the United States. All HPIV3 sequences were grouped into the three major genetic clusters: A, B, and C. Clusters C was further divided into distinct subclusters (C1–C5) and genetic lineages (C1a–C1d and C3a–C3g). The 19 HPIV3 sequences obtained in this study were all grouped into the major sub-cluster C3, within the genetic lineages of C3a and C3f, and were close to the viruses from Malaysia, Japan, and the United States. All the HPIV4 sequences were classified into clades 4A and 4B, with the two sequenced HPIV4 strains (BCH4237A/2014 and BCH4263A/2014) belonging to clade 4A, and close to strains from Malaysia, Japan, Taiwan (China), United States, and India. Phylogenetic analyses revealed that HPIV1–4 co-circulated in China. At the same time, the C3a and C3f lineages of HPIV3 also co-circulated in China. Besides, the estimated values of evolutionary distance and standard error for HPIV3 are shown in Supplementary Table 3. Among them, the divergence values for genetic lineages C3a–C3g were relatively small, ranging from 0.011 ± 0.002 to 0.025 ± 0.003.

A major difference was found between the HN tree and F tree of HPIV3. In the HPIV3 HN tree, BCH4102A/2014 was grouped into the same lineage with BCH4210A/2014, PUMCH14028/2014, and PUMCH13988/2014 and separated from BCH4138A/2014. Conversely, in the HPIV3 F tree, BCH4102A/2014 was grouped into the same lineage with BCH4138A/2014 and separated from BCH4210A/2014, PUMCH14028/2014, and PUMCH13988/2014. This distinction may indicate the existence of a recombination signal in HPIV3.

### Recombination Analysis

**FIGURE 3:**
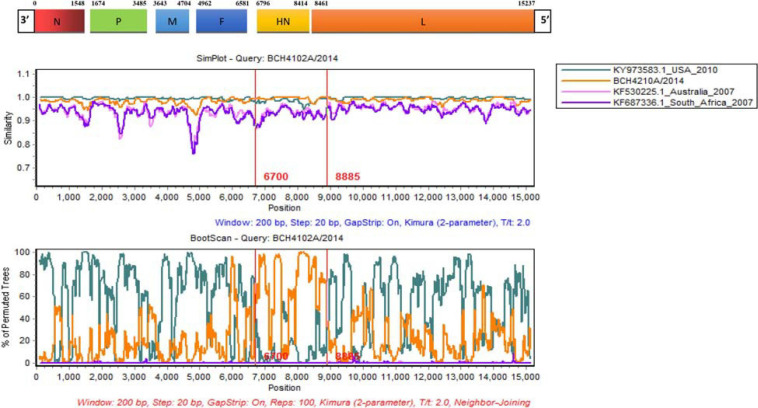
Similarity and bootscaning analyses for complete CDS region of HPIV3 (BCH4102A/2014). The potential recombinant event was detected and the region spanned the HN gene.

Alignments of HPIV1–4 were further used to explore the recombination signal. No recombination event was detected in the newly generated HPIV1, HPIV2, and HPIV4A sequences. For the obtained HPIV3 sequences, a potential recombination signal was predicted in BCH4102A/2014. Two sequences (KY973583 and MW575665) with a high nucleotide similarity and two sequences (KF530225 and KF687336) with a low nucleotide similarity with BCH4102A/2014 were used to perform recombination analysis. Recombination plots and breakpoints of the HN gene of HPIV3 are depicted in Figure 3. The breakpoint locations were at positions 6700 and 8885, and the corresponding region spanned the HN gene. KY973583 (United States) was presumed to be the major parent, as the sequence had the highest nucleotide similarity (99.5%) with the query sequence at the level of the whole CDS region. BCH4210A/2014 identified herein was deduced to be the minor parent because the sequence had the highest nucleotide similarity (99.8%) with the query sequence at the region of the complete HN gene.

### Glycosylation Site Analysis

For the F protein of HPIV1, one potential *N*-glycosylation site (amino acid position 241) and three potential *O*-glycosylated sites (amino acid positions 102, 103, and 453) were predicted. For the HN protein of HPIV1, *N*-glycosylation sites were predicted at amino acid positions 19, 173, 277, 361, 499, and 504, and *O*-glycosylation sites were predicted at amino acid positions 79 and 151.

Five potential N-linked glycosylation sites (amino acid positions 65, 69, 77, 90, and 431) were identified in the F protein of the two obtained HPIV2 sequences, consistent with most sequences available in GenBank. Conversely, no *O*-glycosylated site was found in the F protein of the two viruses identified as HPIV2. The *N*-glycosylated sites (amino acid positions 6, 272, 284, 316, 335, 341, 454, 501, and 517) in the HN amino acid sequences between the two identified HPIV2 viruses were consistent, however, the *O*-glycosylated sites differed between WH17030d1/2017 (amino acid positions 325, 326, 332, 343, 348, and 442) and WH17080d3/2017 (amino acid positions 325, 326, 343, 442, and 446).

For 10 sequences of the F protein of HPIV3, four N-linked glycan sites (amino acid positions 238, 359, 446, and 508) and two (amino acid positions 244 and 246) O-linked glycan sites were predicted. Among the 19 new HPIV3 sequences, the potential *N*-glycosylated sites (amino acid positions 308, 485, and 523) in the HN protein were identical, except that there was no *N*-glycosylated site at amino acid residue 485 in one HPIV3 (BCH4102A/2014). In addition, the N485 site of the HN protein of HPIV3 is unlikely to be glycosylated due to conformational constraints (Asn-Pro-Thr). The deduced *O*-glycosylated sites (amino acid positions 126, 143, 352, 353, and 359) were identical among the 19 obtained HPIV3 sequences.

Three potential *N*-glycosylation sites (amino acid positions 66, 74, and 244) were identified in the F protein of the two obtained HPIV4A sequences, but no *O*-glycosylated site was found. The HN protein of two HPIV4A were predicted to have *N*-glycosylation sites at amino acid positions 279, 339, 347, 433, 502, and 530 and *O*-glycosylated sites at amino acid positions 122, 124, 332, and 348.

### Selective Pressure Analysis

We analyzed the positive and negative selection sites in the 10 F gene sequences and 19 HN gene sequences of HPIV3 identified in this study, because the sequences of the newly identified HPIV1, HPIV2 and HPIV4 viruses were limited. No positive selection sites were found by two methods, whereas 14 and 6 negative selection sites were estimated in the 10 F gene sequences and 19 HN gene sequences by the FEL method, respectively. The details of these analyses are listed in Table 4.

**TABLE 4:** Positive and negative selection sites in the F and HN genes of identified HPIV3.

**Gene**	**Amino acid position^*a*^**	**MEME (positive)**	**FEL (positive/negative)**
F	16 **108 121 154 175 210 262 278 318 365 374 398 483** 533	No	No/Yes (14)
HN	22 38 51 **227 503 513**	No	No/Yes (6)

**FIGURE 4:**
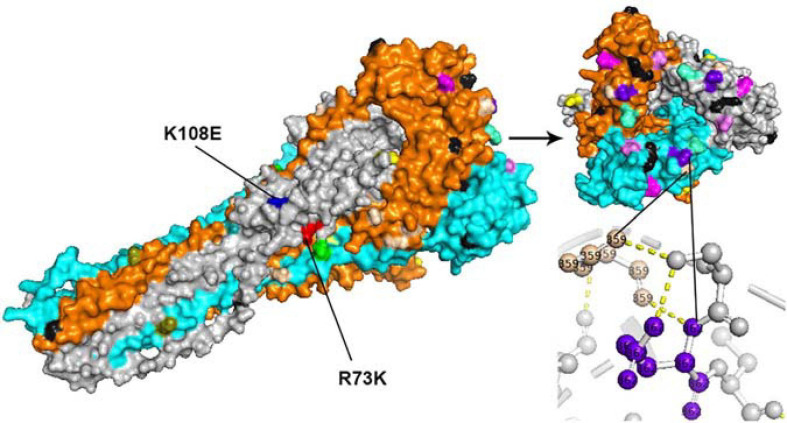
The amino acid substitution sites, negative selection sites and glycosylated sites in the 10 F proteins of obtained HPIV3 were mapped on the structure model of the prototype strain Wash/47885/57. The template for homology modeling was using the crystal structure of 1ZTM. Chains of the trimeric structure model were colored in gray (chain A), orange (chain B), and cyan (chain C). The negative selection sites and glycosylated sites were shown as spheres in black and wheat-colored, respectively. And the remaining colorful spheres correspond to amino acid substitution sites.

**FIGURE 5:**
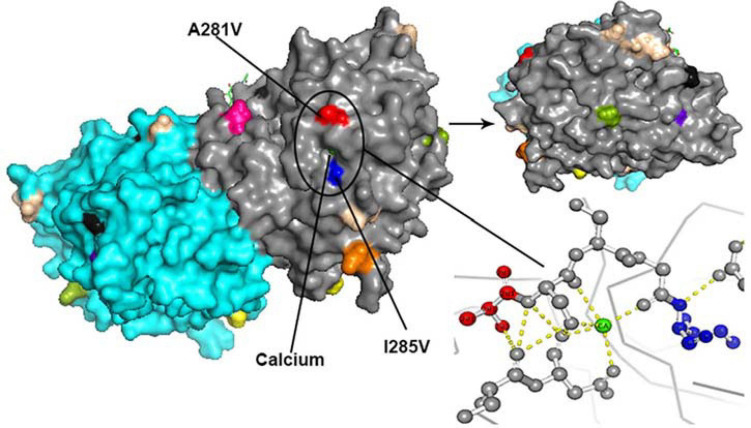
The amino acid substitution sites, the negative selection sites and the glycosylated sites in the 19 HN proteins of obtained HPIV3 were mapped on the structure model of prototype strain Washington 1957. Template for homology modeling was using the crystal structure of 4MZE. Chains of the dimer structure model were colored in gray (chain A) and cyan (chain B). The negative selection sites and glycosylated sites were shown as spheres in black and wheat-colored, respectively. And the remaining colorful spheres correspond to amino acid substitution sites.

*^*a*^The site with bold font was used to mapped on the crystal structures in Figures 4, 5.*

### Amino Acid Substitutions, Negative Selection Sites, and MAb-Binding Sites Mapped on the F and HN Proteins of HPIV3

All amino acid substitutions in the F and HN proteins of the identified HPIV1–4 are listed in Supplementary Table 4. Herein, we mapped the amino acid substitution sites, the negative selection sites and the glycosylated sites in the F and HN protein of HPIV3 on crystal structures (Figures 4, 5). In 10 sequences of the F protein of HPIV3, an amino acid substitution R73K corresponding to a reported monoclonal antibody (MAb)-binding site (Coelingh and Winter, 1990) occured in PUMCH14028/2014, which was classified into C3a. In 19 sequences of the HN protein of HPIV3, an amino acid substitution A281V corresponding to a reported neutralization-related site (Coelingh et al., 1986; van Wyke Coelingh et al., 1987) occured in PUMCH13961/2014, which belonged to C3f. In addition, one negative selection site (amino acid position 398) in the F protein of HPIV3 corresponded to the reported Mab-binding site (Coelingh and Winter, 1990). Moreover, amino acid substitution site (K108E) corresponded to the negative selection site (amino acid position 108) in the F protein of obtained HPIV3. However, no amino acid substitution site corresponded to the glycosylation site in the identified HPIV3.

In the three-dimensional structure model of the F protein of HPIV3, a hydrogen bond between amino acid substitution site Q362R and *N*-glycosylation site N359 occured in BCH4100A/2014, which belonged to C3a. In the three-dimensional structure model of the HN protein of HPIV3 (PUMCH13961/2014), two amino acid residues (A281V and I285V) were closed to the calcium, which might be benefit for conformation in this region.

## Discussion

In this study, we first used the traditional method, Sanger sequencing, to obtain the viral genomes of eight HPIV-positive specimens (HPIV1, HPIV2, and HPIV3). However, due to the low viral load, it was difficult to obtain the complete CDS. Further, the process was labor-intensive and time-consuming. Thus, we designed six sets of universal primers to amplify the whole genomes of 16 HPIV-positive specimens (HPIV3 and HPIV4) and performed NGS. The length of the amplified fragment of each set of primer was about 2–3 kb, and the overlap exceeded 200 bp.

A total of 24 HPIV-positive samples were collected from patients of different ages in Beijing, Chengdu, Xian, Wuhan, Jilin, and Harbin in China. The samples were collected at different months, which indicated that people were susceptible to the virus throughout the year. Table 1 shows that clinical infections were mainly caused by HPIV3, and the clinical manifestations of HPIV1 and HPIV2 infections were milder than those of HPIV3 and HPIV4 infections. The identified sequences expanded the sequence information of HPIV1–4, but there is still a lack of a complete CDS for the Chinese HPIV4B. The overall similarities of the nucleotide and amino acid identities (91.3–99.5%) showed that the HPIV1–4 genomes were relatively stable with less than 8.7% gene mutations.

Phylogenetic analyses showed that different genetic lineages of HPIV3 in sub-cluster C3 co-circulated in China, with C3f as the dominant lineage according to the HN gene sequence. Based on the phylogenetic tree of the HN gene of HPIV2 in a previous study (Santak et al., 2018), G1c was defined as a new sub-cluster in this study. Two HPIV4 sequences belonged to cluster 4A, and cluster 4B was not found in this study. One HPIV1 was classified into clade 2 in the F and HN phylogenetic trees. This results showed that the identified HPIV1–4 co-circulated in China and were genetically closed to viruses from the United States, South Korea, Japan, Malaysia, Vietnam, India, Australia, and Mexico. This study indicated that HPIVs were not limited to a certain country, consistent with a previous work that showed three levels of transmission for HPIV3: local, regional, and global (Goya et al., 2016). Overall, the genetic classification of HPIV3 based on the HN gene phylogenetic tree was consistent with the classification by Aso et al. (2020), but slightly different from that by other molecular epidemiological studies (Mao et al., 2012; Almajhdi, 2015; Goya et al., 2016; Kosutic-Gulija et al., 2017; Jornist et al., 2018). Of the 24 viral sequences verified in this study, no sequences were found to belong to a novel cluster, clade, or lineage, except WH17080d3/2017, indicating that HPIVs have a relatively stable transmission within certain geographical areas. Notably, the distribution of the identified BCH4102A/2014 was different between the HN and F trees, which indicated a potential recombination signal.

RNA viruses have a high genetic variation through mutation and recombination in replication due to viral RNA dependent RNA polymerases (Dolan et al., 2018). Although recombination events are more common in positive-sense RNA viruses, they have been found in negative-sense RNA viruses from the families Paramyxoviridae (RSV and Newcastle disease virus) and Bunyaviridae (Tula hantavirus) (Plyusnin et al., 2002; Chare et al., 2003; Spann et al., 2003; Rui et al., 2010; Bentley and Evans, 2018). This study performed recombination analysis according to the CDS of HPIV1–4, and a potential recombination event was only found in HPIV3. The virus KY973583 from the United States identified in 2010 was presumed to be the major parent because it had the highest similarity (99.5%). Viruses from Peru identified in 2007 (KJ672606) and the United States identified in 2011 (MF973185 and MF973189) were also deduced to be major parents at the whole CDS level, because they had similarities of 99.4% with BCH4102A/2014. Another two consensus sequences (PUMCH14028/2014 and PUMCH13988/2014) have been deduced as minor parents because they had similarities of 99.6% at the complete HN gene sequence level. Nevertheless, these recombination signals were not obvious in the region of the complete HN gene. These results indicated that the HPIV3 prevalent in China in 2014 had undergone slight genetic mutations and was gradually becoming close to viral strains from the Americas.

Glycosylation is an important posttranslational modification that influences protein folding, antigenicity, and biological activity, and the F and HN proteins of HPIVs are closely related to viral infection (Yao et al., 1997; Komada et al., 2000; Chu et al., 2013; Steentoft et al., 2013). Our results showed that neither HPIV2 nor HPIV4 had *O*-glycosylated sites and the *N* and *O*-linked glycosylation sites of the F and HN genes in HPIV1–3 are mostly consistent with those previously reported, except for some sites (Kosutic-Gulija et al., 2016, 2017; Santak et al., 2016). For example, the *N*-glycosylation sites in HN protein of obtained HPIV3 were identical to HPIV3 strains from Kenya at amino acid positions 308, 485, and 523 (Elusah et al., 2020), whereas the *N*-glycosylation site at the position N351 was identified in the HN protein of HPIV3 from Argentina, Japan, and Croatia (Goya et al., 2016; Kosutic-Gulija et al., 2017; Takahashi et al., 2018). The *N*-linked glycans at residue 173 of the HN protein of HPIV1, and at residue 523 of the HN protein of HPIV3, have been considered to mask a second receptor-binding site (Alymova et al., 2008; Mishin et al., 2010).

Due to the length of the templates used for homology modeling, some sites were not mapped on the crystal structures, including an *N*-glycosylated site (amino acid position 508) in the F protein of HPIV3, and an *O*-glycosylated site (amino acid position 126) in the HN protein of HPIV3, and some sites in Table 4 and in Supplementary Table 4. In the obtained HPIV3 sequences (10 F gene and 19 HN gene sequences), we found two amino acid substitution sites and one negative selection sites that corresponded to neutralization-related sites that suggested in previous studies. An amino acid substitution in the F protein at position R73K, responsible for the neutralization-resistance to site B MAbs, was found to naturally occur in only one identified HPIV3 (PUMCH14028/2014) belonging to C3a, which might be associated with viral reinfection in children (Coelingh and Winter, 1990). Meanwhile, an amino acid substitution (A281V) related to mouse MAb-binding site (Coelingh et al., 1986; van Wyke Coelingh et al., 1987; Takahashi et al., 2018) was found in the HN protein of another HPIV3 (PUMCH13961/2014) that belonged to C3f. Although the amino acid substitution site N485S was found in the HN protein of BCH4102A/2014, no *N*-glycosylated site was found in the amino acid residue 485 of BCH4102A/2014. Therefore, no amino acid substitution site corresponded to the glycosylation site in the obtained HPIV3 sequences.

In summary, the twenty-four consensus sequences of HPIV1–4 obtained in this study provide a basis for an in-depth analysis of the spread, recombination, and mutation of the viruses. Furthermore, these results may promote virus diagnosis and vaccine development, and aid in monitoring and prevention. However, there is still a demand for further epidemiological studies to collect sequence information for HPIV1–4 from different regions and age groups to understand the molecular evolution of the viruses circulating in China.

## Data Availability Statement

The datasets presented in this study can be found in online repositories. The data presented in the study are deposited in the GenBank database (accession numbers: MW575643–MW575666), and the NCBI sequence read archive (SRA) under accession number PRJNA701861 (SAMN17915237–SAMN17915250).

## Ethics Statement

Written informed consent was obtained from the individual(s), and minor(s)’ legal guardian/next of kin, for the publication of any potentially identifiable images or data included in this article.

## Author Contributions

TZ, FY, and NS designed the project. YX, XW, and LR collected the samples. NS, TZ, JD, LS, and YZ conducted the experiments. NS, FY, TZ, and BL analyzed the data. NS wrote the manuscript. All authors read and approved the submitted version.

## Conflict of Interest

The authors declare that the research was conducted in the absence of any commercial or financial relationships that could be construed as a potential conflict of interest.

## Footnotes

1 http://web.cbio.uct.ac.za/∼darren/rdp.html

2 https://sray.med.som.jhmi.edu/SCRoftware/

3 https://services.healthtech.dtu.dk/

4 http://datamonkey.org/

5 SWISS-MODEL is a fully automated protein structure homology-modelling server available at https://swissmodel.expasy.org

## Supplementary Material

The Supplementary Material for this article can be found online at Figshare via the doi: 10.6084/m9.figshare.14508543.v1.

Supplementary Table 1 Universal primers for full-length amplification of 16 HPIV-positive specimens.

Supplementary Table 2 Estimates of evolutionary divergence over sequence pairs between groups of the entire HPIV3 F gene.

Supplementary Table 3 Estimates of evolutionary divergence over sequence pairs between groups of the entire HPIV3 HN gene.

Supplementary Table 4 Amino acid substitution sites in the F and HN proteins of HPIV1–4 obtained in this study.
