# Supplementary material for: Genetic Characteristics of Human Parainfluenza Virus Types 1–4 From Patients With Clinical Respiratory Tract Infection in China
Source: Front Microbiol. 2021 Jul 15;12:679246. doi: 10.3389/fmicb.2021.679246 (PMC8320325; doi:10.3389/fmicb.2021.679246)
Supplement: Supplementary Table 1 — Universal primers for full-length amplification of 16 HPIV-positive specimens. [file Data_Sheet_1.ZIP › Supplementary Table 2.DOC]

**Supplementary TABLE 2.** Estimates of evolutionary divergence over sequence pairs

between groups of the entire HPIV3 F gene

| Groups | A | B | C1a | C1b | C2 | C3a | C3b | C5 |
| --- | --- | --- | --- | --- | --- | --- | --- | --- |
| A |  | 0.006 | 0.007 | 0.007 | 0.007 | 0.007 | 0.007 | 0.007 |
| B | 0.052 |  | 0.006 | 0.006 | 0.006 | 0.006 | 0.006 | 0.006 |
| C1a | 0.056 | 0.058 |  | 0.002 | 0.005 | 0.004 | 0.005 | 0.005 |
| C1b | 0.059 | 0.063 | 0.014 |  | 0.005 | 0.005 | 0.005 | 0.005 |
| C2 | 0.052 | 0.058 | 0.031 | 0.036 |  | 0.003 | 0.003 | 0.003 |
| C3a | 0.057 | 0.063 | 0.034 | 0.036 | 0.020 |  | 0.002 | 0.004 |
| C3b | 0.055 | 0.061 | 0.034 | 0.039 | 0.018 | 0.018 |  | 0.003 |
| C5 | 0.056 | 0.065 | 0.038 | 0.042 | 0.016 | 0.029 | 0.026 |  |
