# Supplementary material for: Genetic Characteristics of Human Parainfluenza Virus Types 1–4 From Patients With Clinical Respiratory Tract Infection in China
Source: Front Microbiol. 2021 Jul 15;12:679246. doi: 10.3389/fmicb.2021.679246 (PMC8320325; doi:10.3389/fmicb.2021.679246)
Supplement: Supplementary Table 1 — Universal primers for full-length amplification of 16 HPIV-positive specimens. [file Data_Sheet_1.ZIP › Supplementary Table 3.DOC]

**Supplementary TABLE 3.** Estimates of evolutionary divergence over sequence pairs between groups of the entire HPIV3 HN gene

| Groups | A | B | C1a | C1b | C1c | C1d | C2 | C3a | C3b | C3c | C3d | C3e | C3f | C3g | C4 | C5 |
| --- | --- | --- | --- | --- | --- | --- | --- | --- | --- | --- | --- | --- | --- | --- | --- | --- |
| A |  | 0.005 | 0.006 | 0.006 | 0.006 | 0.006 | 0.006 | 0.005 | 0.005 | 0.006 | 0.006 | 0.005 | 0.006 | 0.005 | 0.004 | 0.006 |
| B | 0.044 |  | 0.005 | 0.006 | 0.006 | 0.006 | 0.005 | 0.006 | 0.005 | 0.005 | 0.006 | 0.005 | 0.006 | 0.006 | 0.004 | 0.006 |
| C1a | 0.055 | 0.054 |  | 0.003 | 0.003 | 0.002 | 0.004 | 0.004 | 0.004 | 0.005 | 0.004 | 0.004 | 0.004 | 0.004 | 0.004 | 0.005 |
| C1b | 0.053 | 0.052 | 0.020 |  | 0.004 | 0.003 | 0.004 | 0.005 | 0.004 | 0.005 | 0.005 | 0.004 | 0.005 | 0.005 | 0.005 | 0.005 |
| C1c | 0.056 | 0.055 | 0.022 | 0.023 |  | 0.003 | 0.005 | 0.005 | 0.005 | 0.005 | 0.005 | 0.005 | 0.005 | 0.005 | 0.005 | 0.005 |
| C1d | 0.053 | 0.051 | 0.016 | 0.016 | 0.018 |  | 0.004 | 0.004 | 0.004 | 0.004 | 0.004 | 0.004 | 0.004 | 0.004 | 0.004 | 0.004 |
| C2 | 0.055 | 0.053 | 0.038 | 0.039 | 0.042 | 0.036 |  | 0.003 | 0.003 | 0.004 | 0.004 | 0.003 | 0.003 | 0.004 | 0.004 | 0.004 |
| C3a | 0.055 | 0.053 | 0.039 | 0.038 | 0.040 | 0.035 | 0.029 |  | 0.002 | 0.003 | 0.002 | 0.002 | 0.003 | 0.003 | 0.004 | 0.004 |
| C3b | 0.054 | 0.051 | 0.038 | 0.037 | 0.039 | 0.034 | 0.030 | 0.016 |  | 0.003 | 0.002 | 0.002 | 0.002 | 0.002 | 0.004 | 0.004 |
| C3c | 0.052 | 0.053 | 0.041 | 0.040 | 0.042 | 0.037 | 0.031 | 0.024 | 0.023 |  | 0.003 | 0.003 | 0.003 | 0.003 | 0.005 | 0.004 |
| C3d | 0.054 | 0.055 | 0.040 | 0.039 | 0.042 | 0.036 | 0.031 | 0.011 | 0.018 | 0.025 |  | 0.002 | 0.003 | 0.003 | 0.004 | 0.004 |
| C3e | 0.052 | 0.050 | 0.036 | 0.035 | 0.037 | 0.032 | 0.028 | 0.013 | 0.015 | 0.022 | 0.015 |  | 0.002 | 0.002 | 0.004 | 0.004 |
| C3f | 0.054 | 0.053 | 0.040 | 0.038 | 0.040 | 0.036 | 0.031 | 0.017 | 0.018 | 0.025 | 0.019 | 0.015 |  | 0.003 | 0.004 | 0.004 |
| C3g | 0.054 | 0.053 | 0.038 | 0.037 | 0.038 | 0.034 | 0.029 | 0.015 | 0.015 | 0.022 | 0.016 | 0.014 | 0.017 |  | 0.004 | 0.004 |
| C4 | 0.027 | 0.023 | 0.031 | 0.031 | 0.033 | 0.029 | 0.032 | 0.032 | 0.033 | 0.035 | 0.033 | 0.030 | 0.032 | 0.032 |  | 0.004 |
| C5 | 0.053 | 0.052 | 0.040 | 0.035 | 0.042 | 0.034 | 0.029 | 0.024 | 0.025 | 0.027 | 0.025 | 0.024 | 0.027 | 0.024 | 0.033 |  |
