# Supplementary material for: Genetic Characteristics of Human Parainfluenza Virus Types 1–4 From Patients With Clinical Respiratory Tract Infection in China
Source: Front Microbiol. 2021 Jul 15;12:679246. doi: 10.3389/fmicb.2021.679246 (PMC8320325; doi:10.3389/fmicb.2021.679246)
Supplement: Supplementary Table 1 — Universal primers for full-length amplification of 16 HPIV-positive specimens. [file Data_Sheet_1.ZIP › Supplementary Table 1.DOC]

**Supplementary TABLE 1.** Universal primers for full-length amplification of 16 HPIV-positive specimens

| Primers | Sequences of primers(5′-3′) | First thermal profile | Second thermal profile | Product Size (bp) |
| --- | --- | --- | --- | --- |
| HPIV3.F13 | 5'CTCTAYAATTTCAAAAATGTTGAGC | 94°C for 5 min;  94°C for 30 s,  50°C for 30 s,  72°C for 3min,  30 cycles. 72°C for 10 min | 94°C for 5 min;  94°C for 30 s,  50°C for 30 s,  72°C for 3min,  40 cycles. 72°C for 10 min | 3081 |
| HPIV3.R3093 | 5'AAGATTTGAAATTAATGACGTGATC |  |  |  |
| HPIV3.F2726 | 5'AGACACAAAAGACAAATGGAAAGG |  |  | 3219 |
| HPIV3.R5944 | 5'TCGTCATGATATGGCTGGGAAG |  |  |  |
| HPIV3.F5680 | 5'ATAGCATCATTATACCGCAC |  |  | 3179 |
| HPIV3.R8858 | 5'ACATTTCTTTTGACATTTCTGG |  |  |  |
| HPIV3.F8595 | 5'CTGACATACTCTATCCTGAGTG |  |  | 3159 |
| HPIV3.R11753 | 5'GAATATCATGTGCAACTCTAGG |  |  |  |
| HPIV3.F11212 | 5'TTCACCTGTTCTAGGATATGC |  |  | 3107 |
| HPIV3.R14318 | 5'GTAGAACAGTTTCTTCTGATTTACC |  |  |  |
| HPIV3.F12123 | 5'ACCCATATACTTGGATGTATTTAC |  |  | 3108 |
| HPIV3.R15230 | 5'TTTCTAATAACTGTTCTTCGGG |  |  |  |
|  |  |  |  |  |
| HPIV4-F3 | 5'CAAGGGGAGAAGAGATATRG | 94°C for 5 min;  94°C for 30 s,  50/42/58/50/50/42 °C ^c^ for 30 s,  72°C for 3min,  30 cycles. 72°C for 10 min | 94°C for 5 min;  94°C for 30 s,  50/42/58/50/50/42 °C ^c^  for 30 s,  72°C for 3min,  40 cycles. 72°C for 10 min | 3095 |
| HPIV4.R3097 | 5'AAGTGCATCTATACGAACACC |  |  |  |
| HPIV4.F2997 | 5'AAGACAATACAATTACACTTG |  |  | 3072 |
| HPIV4.R6068 | 5'TATTTGTGTGAATTGTTATAG |  |  |  |
| HPIV4.F5528 | 5'GCTGCACARGTTACAGCAGCAATCG |  |  | 3245 |
| HPIV4.R8772 | 5'GGCCACCAGCTGGATCCTCTC |  |  |  |
| HPIV4.F8579 | 5'TCATACACAATAACTTACTTCTC |  |  | 3078 |
| HPIV4.R11656 | 5'ATATTCTCCCAGTTTCTTTTATC |  |  |  |
| HPIV4.F10954 | 5'TTACATGATGTTTTCTCAGTTG |  |  | 3418 |
| HPIV4.R14011 | 5'CATTGCATTCATCAATTTGTAGC |  |  |  |
| HPIV4.F14649 | 5'AAGAAGAATGATTAATTTAGG |  |  | 2340 |
| HPIV4.R16988 | 5'TGTTTKWWWTTTTTTATTAAAC |  |  |  |

^c^The six annealing temperatures corresponded to the six sets of primers used for PCR amplification.
