# Supplementary material for: Genetic Characteristics of Human Parainfluenza Virus Types 1–4 From Patients With Clinical Respiratory Tract Infection in China
Source: Front Microbiol. 2021 Jul 15;12:679246. doi: 10.3389/fmicb.2021.679246 (PMC8320325; doi:10.3389/fmicb.2021.679246)
Supplement: Supplementary Table 1 — Universal primers for full-length amplification of 16 HPIV-positive specimens. [file Data_Sheet_1.ZIP › Supplementary Table 4.doc]

**Supplementary TABLE 4.** Amino acid substitution sites in the F and HN proteins

of HPIV1–4 obtained in this study

| Genotype | Gene | Prototype strain  (accession number) | Virus | Amino acid substitution site^d^ |
| --- | --- | --- | --- | --- |
| HPIV1 | F | Washington 1964  (AF457102) | JL18058d5/2018 | K3S L7F F8L V25I E26D V155I L163F A214T T249I N321S R410K V500I H532N V536I N553H |
|  | HN | Washington 1964  (AF457102) | JL18058d5/2018 | N8I V22A T28I G31R H34Y T45A V46A F49L I59V M66I M76V I82T R131K S151T I245V I335V D349N N355D R356S T358P N443K K448D E453K O461P R468E V489F N511S V524E E527K A553T I573V |
| HPIV2 | F | GREER  (AF533012) | WH17030d1/2017 | D22G T96A R163K N247K K289R R390K S524A |
|  |  |  | WH17080d3/2017 | I32V T96A T102P Q104R V112I V158L D160T I165T A166V N247K R390K S524A F538V |
|  | HN | GREER  (AF533012) | WH17030d1/2017 | D54N I67V Y100L T117S K139N N164H I175S E254D K316N P319S N322K K323E K332T K341N Q345R A348T S351G N360Y V367I H376Q N381S A416S P479L R497K S513N A514S |
|  |  |  | WH17080d3/2017 | V48F S49P D57E S69L Y100L T114A I132V L138P K139E I175S T195A A201S A211G V242I E254D S281F K316N P319T K323E K341N E344R A347E A348I M375I R379E A416S P446T D476N P479I T480M Q482R R497K A521T S513N A514S P571L |
| HPIV3 | F | Wash/47885/57  (S82195) | BCH4100A/2014 | P2L T3I L7S F17H **S95T K108E I165V Q362R V367T N441D** **K462R** N486S I496V V497I I501M V509I I513T V516I |
|  |  |  | BCH4102A/2014 | T3I F17H **S95T K108E V367T** N486S I496V V497I I501M V509I I513T V516I |
|  |  |  | BCH4210A/2014 | P2L T3I L7S F17H **S95T K108E I165V V367T N441D** N486S I496V V497I I501M V509I I513T V516I |
|  |  |  | PUMCH12693/2012 | P2L T3I L7S F17H **S95T K108E I165V V367T N441D** N486S H488Y I496V V497I I501M V509I I513T V516I |
|  |  |  | PUMCH13988/2014 | P2L T3I L7S F17H **S95T K108E I165V V367T N441D** N486S I496V V497I I501M V509I I513T V516I |
|  |  |  | PUMCH14028/2014 | P2L T3I L7S F17H **R73K S95T K108E I165V V367T N441D** N486S I496V V497I I501M V509I I513T V516I |
|  |  |  | BCH3297A/2012 | P2L T3I L7S F17H **S95T K108E I165V V367T N441D S448F** N486S I496V V497I I501M V509I I513T V516I |
|  |  |  | BCH4138A/2014 | T3I F17H **S95T** **K108E V367T N441D** N486S I496V V497I I501M V509I I513T V516I |
|  |  |  | BCH3242A/2012 | P2L T3I L7S M14V F17H **H27R S95T K108E I165V V367T N441D** N486S I496V V497I I501M V509I I513T V516I K532Q |
|  |  |  | BCH4066A/2014 | T3N F17H **S95T K108E H206Y H350Y V367T N441D** N486S I496V V497I I501M V509I I513T V516I |
|  | HN | Washington 1957  (JN089924) | BCH4100A/2014 | M21T N26S I28L I40T I53T S58N H62R V69I E78G M118I **H295Y 1391V D556N** |
|  |  |  | BCH4102A/2014 | M21T N26S I28L I40T I53T H62R V69I E78G E78G M118I **H295Y 1391V N485S D556N** |
|  |  |  | BCH4210A/2014 | M21T N26S I28L I40T I53T H62R V69I E78G M118I **H295Y 1391V D556N** |
|  |  |  | PUMCH12693/2012 | M21T N26S I28L I40T I53T H62R V69I E78G M118I **H295Y 1391V D556N** |
|  |  |  | PUMCH13988/2014 | M21T N26S I28L I40T I53T H62R V69I E78G M118I **H295Y 1391V R446K D556N** |
|  |  |  | PUMCH14028/2014 | M21T N26S I28L I40T I53T H62R V69I E78G M118I I130N **H295Y 1391V D556N** |
|  |  |  | BCH3297A/2012 | M21T N26S I28L I40T I53T H62R V69I E78G M118I **H295Y 1391V D556N** |
|  |  |  | BCH4138A/2014 | M21T I28L I40T I53T H62R V69I H295Y 1391V D556N |
|  |  |  | BCH3242A/2012 | H9Q M21T N26S I28L I40T I53T S58N H62R V69I E78G M118I **H295Y 1391V D556N** |
|  |  |  | BCH4066A/2014 | E16G M21T I28L I40T I53T H62R V69I E78G M118I **H295Y D556N** |
|  |  |  | PUMCH12726/2012 | M21T N26S I28L I40T I53T H62R V69I E78G M118I **I285V H295Y 1391V D556N** |
|  |  |  | PUMCH12885/2012 | G14S M21T I28L I40T I53T H62R V69I E78G M118I **Q222P H295Y 1391V D556N** |
|  |  |  | PUMCH13961/2014 | M21T N26S I28L I40T I53T H62R V69I E78G M118I **A281V I285V H295Y 1391V D556N** |
|  |  |  | BCH3283A/2012 | M21T N26S I28L I40T I53T H62R V69I E78G M118I **H295Y 1391V D556N** |
|  |  |  | HB16067d9/2017 | M21T I28L I40T I53T H62R V69I M118I **H295Y 1391V D556N** |
|  |  |  | WH17001d3/2017 | G14S M21T I28L I40T I53T H62R V69I E78G M118I **H295Y 1391V D556N** |
|  |  |  | XA17013d1/2017 | M21T I28L I40T I53T H62R V69I E78G M118I **H295Y 1391V D556N** |
|  |  |  | CD17001d7/2017 | M21T I28L I40T I53T H62R V69I E78G M118I **H295Y 1391V D556N** |
|  |  |  | CD17004d3/2017 | E16G M21T I28L I40T I53T H62R V69I E78G M118I **H295Y 1391V D556N** |
| HPIV4A | F | M-25  ( AB54336) | BCH4237A/2014 | L6S I23V K93Q I100V P231S F310V E341V T440S T503I Y533Q K534R |
|  |  |  | BCH4263A/2014 | L6S I23V I100V F105V P231S F310V E341V Y533Q K534R |
|  | HN | M-25  ( AB54336) | BCH4237A/2014 | D3E S4L G6S L11F G27R I59T K62R S121G L125V G127R N137K F139I T166A P200T S202H M217N R234K G238S R250L N251Y T254I L266P N267Q W268L Q269A H270T N271Q Y272L H274T T275Y Y276I H277S I278Y M279N V280G P281T L282I T284D L307I K311N L312Y L313I M350V L379I Q381R K436N R440K I478T L479W Q493R I555L T568N L569P A572R A573L Q574I |
|  |  |  | BCH4263A/2014 | G6S L11F G27R I59T S121G L125M G127R N137K F139I T166A T166A P200T S202H M217N R234K G238S R250L N251Y T254I L266P N267Q W268L Q269A H270T N271Q Y272L H274T T275Y Y276I H277S I278Y M279N V280G P281T L282I T284D L307I K311N L312Y L313I M350V Q381R K436N R440K I478T L479W Q493R I555L T568N L569P A572R A573L Q574I |

^d^The site with bold font was used to mapped on the crystal structures in Figure 4 and Figure 5
